# Editorial: The dynamics in global malnutrition uncovered

**DOI:** 10.3389/fpubh.2026.1974020

**Published:** 2026-10-07

**Authors:** Phillips Edomwonyi Obasohan, Joseph P. Mensah, Robert Akparibo

**Affiliations:** 1 Department of Liberal Studies, College of Administrative and Business Studies, Niger State Polytechnic, Bida, Nigeria; 2 School of Medicine and Population Health, University of Sheffield, Sheffield, United Kingdom; 3 Nuffield Department of Primary Care Health Sciences, University of Oxford, Oxford, United Kingdom

**Keywords:** double burden of malnutrition (DBM), malnutrition, micronutrients, undernutrition, overnutrition, overweight, obesity, multiple burden of malnutrition (MBM)

## Introduction

Over the past two decades, malnutrition has evolved into a complex global health challenge. More than 2 billion people are affected by micronutrient deficiencies (1), 2.5 billion adults are overweight or living with obesity (2), and more than 150 million children under the age of five are stunted (3, 4). Increasing attention is also being given to the double and triple burdens of malnutrition, in which undernutrition coexists with overweight and obesity and, in some contexts, micronutrient deficiencies within the same population, household, or individual (5). Key drivers of this changing nutrition profile include the global shift toward ultra-processed foods, early-life metabolic programming, poverty, and food insecurity (6, 7).

In response to the coexistence of malnutrition crises, global health authorities and governments have promoted double-duty actions: policies and interventions designed to address undernutrition and overweight and obesity simultaneously (8). These efforts are increasingly linked to universal health coverage, improved diet quality, and broader food-system transformation (7). Additional strategies include fortification and biofortification, school health programs, regulatory frameworks, and restrictions on the marketing of unhealthy foods (9). Despite these efforts, current evidence suggests that the world remains off track to meet nutrition-related Sustainable Development Goal targets by 2030 (4, 10). However, understanding the dynamics of malnutrition is useful only when responses are applied equitably, effectively, and in ways that reflect population needs (11).

## The aims in focus

The key goals of this Research Topic in Frontiers of Public Health and Nutrition: “*The Dynamics in Global Malnutrition Uncovered*,” are to assess demographic and economic shifts, examine the effects of trade on food systems, and explore integrated responses. Drawing on public health, nutrition, and global health perspectives, the issue aims to develop practical, future-focused solutions to malnutrition. This Research Topic brought together 10 original articles, research perspectives, and a systematic review to explain these complex and interconnected challenges.

## The included papers

Collectively, the selected papers examine global malnutrition as a complex public health challenge shaped by place, time, biology, household conditions, food systems, and social inequality. Across the papers, the central message is that malnutrition cannot be understood or addressed through isolated indicators or broad national averages alone. Instead, effective prevention and response require attention to when children become vulnerable, where risk is concentrated, which groups are most affected, and how overlapping nutritional, social, and health-system factors interact.

Agudelo-Ibáñez et al. demonstrate the value of spatiotemporal analysis for identifying deaths due to malnutrition among children under five in Colombia between 2015 and 2023. Their work shows that knowing the location, timing, and population distribution of malnutrition-related mortality is essential for targeting interventions, allocating limited resources, detecting high-risk areas early, and monitoring progress. Similarly, Moran-Herrera et al. approach child nutritional risk as a multidimensional and spatially uneven phenomenon. By combining anthropometric indicators using *Z*-score matrices and multivariate spatial methods, this approach provides a reproducible framework for identifying provinces where children face coexisting forms of nutritional vulnerability. Socioeconomic inequalities are examined by Tan et al., who show that child undernutrition in Pakistan is concentrated among poorer households, with wealth, maternal education, and region explaining much of the observed inequality. Together, these studies highlight the importance of spatial surveillance and equity-sensitive planning in directing public health action toward communities with the greatest need.

Betha et al. add a life-course perspective by analyzing longitudinal data from the LIFE birth cohort in rural Southern India. Their findings show that malnutrition emerges early in childhood, especially during the first 3 years of life, and is particularly significant among girls and children with low birth weight. This reinforces the need for preventive approaches beginning at birth, rather than waiting until severe malnutrition becomes visible. Makwela and Mashaba extend this prevention focus to the complementary feeding period from 6 to 23 months, a critical developmental window in which inadequate feeding practices may lead to stunting, wasting, and micronutrient deficiencies. Their scoping review of 109 papers and over one million caregiver–child pairs identify caregiver knowledge, maternal education, household resources, cultural practices, food affordability, and access to health services as key determinants. Their findings support locally tailored programs rather than uniform interventions.

At a global level, Peng et al. report substantial reductions in iodine and vitamin A deficiencies among children between 1990 and 2021, but little progress in dietary iron deficiency, particularly in populations with a low sociodemographic index. Tan's systematic review and meta-analysis broaden the discussion by showing how childhood malnutrition often overlaps with rickets, anemia, iron deficiency, vitamin A deficiency, infections, poverty, poor diet quality, and limited healthcare access. This study underscores the public health importance of integrating multiple deficiencies into a unified nutrition model, particularly in areas where anemia and micronutrient deficiencies are common. Octavia and Nirmala further expand the framework by examining Indonesia's triple burden of malnutrition from a Gene-to-Plate food-system perspective, with reference to Peru and South Africa. They argue that weakened food systems, declining nutrient quality, biodiversity loss, and limited dietary diversity contribute to persistent malnutrition, while locally rooted food systems and coordinated multisectoral action can support sustainable improvements and advance SDGs 2 and 3.

The remaining studies demonstrate that malnutrition rarely occurs in isolation. Li et al. quantify the burden of diarrhoeal disease, malaria, measles, respiratory infections, and tuberculosis among children with growth failure, highlighting important differences by age and country and a possible future increase in malaria burden. Cui et al. extend this perspective to adults with cancer and comorbidities, showing substantial overlap between malnutrition, frailty, and sarcopenia. Together, these articles position malnutrition as a multidimensional condition shaped by food systems, social inequalities, early-life exposures, infectious disease, micronutrient disorders, and chronic illness.

Overall, these papers converge on the need for evidence-informed, locally grounded, multisectoral strategies that connect early-life prevention, spatial targeting, micronutrient integration, caregiver support, and food-system reform to reduce childhood malnutrition more effectively.

## Conclusion

The contributions to this Research Topic demonstrate that malnutrition is not a single condition with a single cause or solution. It is shaped by interconnected food-system failures, socioeconomic and territorial inequalities, early-life exposures, micronutrient deficiencies, infectious diseases, and chronic conditions operating across the life course.

Addressing these challenges requires integrated, equity-focused action. Nutrition policies must use disaggregated evidence to identify populations being left behind, strengthen early-life interventions, embed nutrition within routine healthcare, and ensure that food systems deliver affordable, accessible, and nutritious diets. Double- and triple-duty interventions offer an important way forward but must be context-specific, adequately financed, and supported by multisectoral governance and robust monitoring.

With 2030 approaching, fragmented and incremental responses are no longer sufficient. The priority must shift from documenting the multiple burdens of malnutrition to transforming the systems and inequalities that create and sustain them.
